# Respiration Monitoring via Forcecardiography Sensors

**DOI:** 10.3390/s21123996

**Published:** 2021-06-09

**Authors:** Emilio Andreozzi, Jessica Centracchio, Vincenzo Punzo, Daniele Esposito, Caitlin Polley, Gaetano D. Gargiulo, Paolo Bifulco

**Affiliations:** 1 Department of Electrical Engineering and Information Technologies, University of Naples Federico II, Via Claudio, 21, 80125 Napoli, Italy; jessica.centracchio@unina.it (J.C.); vinc.punzo@studenti.unina.it (V.P.); daniele.esposito@unina.it (D.E.); 2 School of Engineering, Design and Built Environment, Western Sydney University, Penrith, NSW 2751, Australia; caitlin.polley@westernsydney.edu.au (C.P.); g.gargiulo@uws.edu.au (G.D.G.); 3 The MARCS Institute, Western Sydney University, Penrith, NSW 2751, Australia

**Keywords:** respiration, forcecardiography, continuous monitoring, force sensor, force-sensitive resistors

## Abstract

In the last few decades, a number of wearable systems for respiration monitoring that help to significantly reduce patients’ discomfort and improve the reliability of measurements have been presented. A recent research trend in biosignal acquisition is focusing on the development of monolithic sensors for monitoring multiple vital signs, which could improve the simultaneous recording of different physiological data. This study presents a performance analysis of respiration monitoring performed via forcecardiography (FCG) sensors, as compared to ECG-derived respiration (EDR) and electroresistive respiration band (ERB), which was assumed as the reference. FCG is a novel technique that records the cardiac-induced vibrations of the chest wall via specific force sensors, which provide seismocardiogram-like information, along with a novel component that seems to be related to the ventricular volume variations. Simultaneous acquisitions were obtained from seven healthy subjects at rest, during both quiet breathing and forced respiration at higher and lower rates. The raw FCG sensor signals featured a large, low-frequency, respiratory component (R-FCG), in addition to the common FCG signal. Statistical analyses of R-FCG, EDR and ERB signals showed that FCG sensors ensure a more sensitive and precise detection of respiratory acts than EDR (sensitivity: 100% vs. 95.8%, positive predictive value: 98.9% vs. 92.5%), as well as a superior accuracy and precision in interbreath interval measurement (linear regression slopes and intercepts: 0.99, 0.026 s (R^2^ = 0.98) vs. 0.98, 0.11 s (R^2^ = 0.88), Bland–Altman limits of agreement: ±0.61 s vs. ±1.5 s). This study represents a first proof of concept for the simultaneous recording of respiration signals and forcecardiograms with a single, local, small, unobtrusive, cheap sensor. This would extend the scope of FCG to monitoring multiple vital signs, as well as to the analysis of cardiorespiratory interactions, also paving the way for the continuous, long-term monitoring of patients with heart and pulmonary diseases.

## 1. Introduction

Monitoring respiration is an important task that plays a key role in several different situations [1,2]. Examples are intensive care units, where vital signs of patients with severe health conditions must be continuously monitored [2]; follow-up of chronic obstructive pulmonary diseases (COPD), where long-term monitoring of patients’ respiration can provide useful information about their pathological condition [3,4]; diagnosis and monitoring of sleep apnea, where nocturnal monitoring of respiration is critical for both detecting and avoiding long breath-holding times [5]; and accident and emergency units, where rapid and unobtrusive monitoring of vital signs is required to assess the actual health conditions of the patient [1]. Moreover, respiration monitoring is also fundamental in case of post-anesthesia respiratory depression, which represents one of the main mortality factors after surgery [5]; in sudden infant and adult death syndromes, where early detection of breathing absence is crucial for reducing the number of deaths [6]; and, most recently, in COVID-19 patient management, where respiration monitoring allows evaluating the pulmonary function [7].

Many techniques have been proposed in the literature for noninvasive respiration monitoring and are based on different physical principles, sensors and instrumentation [1,2]. They can be essentially divided into two main classes: contact-based and contactless methods. Moreover, these methods can be further classified according to the particular physical quantity that is actually measured. Indeed, contact-based techniques include methods that rely on respiratory airflow, respiratory sounds, air temperature, air humidity, air components, chest wall movement analysis, thoracic impedance and modulation of other physiological signals [5,8]. In detail, respiratory airflow-based sensors (e.g., flowmeters, fiber-optic sensors) are able to extract the temporal trend of the air exhaled and inhaled by the subject during the breathing, measuring different physical quantities that exhibit linear or nonlinear relationship with the airflow [9,10,11,12,13,14,15]. Microphones, instead, which represent the most common acoustic sensors, allow the measurement of pressure changes caused by the air turbulences during the breathing acts [16,17,18,19,20]. Air temperature sensors (e.g., thermistors, thermocouples) exploit different physical phenomena to measure the temperature variations of the breathed air [21,22,23,24,25,26], whereas air humidity sensors (e.g., capacitive sensors, resistive sensors, nanocrystal and nanoparticle sensors) provide a measurement of the humidity difference in the inhaled and exhaled air, since the latter is richer in water vapor than the former [27,28,29,30,31,32,33,34]. Besides, air component sensors (e.g., end-tidal O_2_/CO_2_ measurement) measure essentially the variations of oxygen and carbon dioxide concentrations in the air, which allow distinguishing the inhalation and exhalation phases [35,36,37,38]. Sensors based on chest wall movements, instead, are substantially sensitive to the deformations of the thorax (e.g., resistive sensors, capacitive sensors) [39,40,41,42,43,44,45,46,47] and to the movements of the chest and abdomen (e.g., accelerometers, gyroscopes) [48,49,50,51,52]. Electric impedance plethysmography measures the changes in transthoracic impedance caused by the variations of air volume in the lungs involved in the respiratory activity [53,54,55,56]. Finally, referring to the methods based on modulation of other physiological signals, the well-known ECG-derived respiration (EDR) [57,58] measures ECG morphology changes due to sinus arrhythmia, relative movement of heart and electrodes and changes in lung volume, whereas the photoplethysmography (PPG)-derived respiration exploits the modulation in amplitude, baseline and frequency of the PPG signal caused by changes in blood stroke volume, heart rate and tissue blood volume and the variation of its pulse wave width under changes in artery stiffness during the respiratory activity [59,60]. On the other hand, contactless techniques comprise methods based on environmental respiratory sounds (e.g., microphones), air temperature (e.g., thermal cameras), chest wall movements (e.g., radar sensors, marker-based stereophotogrammetric systems, stereoscopic camera sensors) or modulation of other physiological signals (e.g., light intensity measurement, RGB cameras) [8,61,62,63,64,65,66,67,68,69,70,71,72,73,74]. The great part of the instrumentation required by such methods is generally cumbersome and far from being wearable, or even portable, so its use has remained confined to research or clinical settings [1,2].

In the last few decades, wearable systems for respiration monitoring have attracted major attention, as their applications not only range from long-term monitoring of both bedridden and ambulatory patients to the tracking of athletes’ performances during sport activities [4,75,76,77,78] but also include the monitoring of people working under heavy psychophysiological stress conditions, such as pilots, soldiers and surgeons [5]. Indeed, wearable systems provide a simple and unobtrusive solution that helps to significantly reduce patients’ discomfort and to improve reliability of measurements, thus supporting long-term monitoring, as opposed to, e.g., methods based on dry electrodes, which suffer from different drawbacks [79,80]. Bifulco et al. showed the possibility of simultaneously monitoring respiration, seismocardiogram and heart sounds by using a single wideband polyvinylidene fluoride (PVDF) piezo film sensor placed on the sternum of the patient, which can also be easily embedded into continuous monitoring devices [81]. Elfaramawy et al. proposed a real-time low-power wireless monitoring system built using a wearable patch sensor, where the two embedded inertial measurement units allow measuring the movements of both the thorax and the abdominal cavity, to obtain the breathing rate [82]. Al-Halhouli et al. fabricated a wearable and stretchable sensor for continuous respiration monitoring, which is composed of a stretchable circuit, made of silver nanoparticles deposited via inkjet printing technology on a polydimethylsiloxane substrate that is attached to a fabric belt. The sensor extracts the respiration rate by detecting the change in the inductance of the conductive pattern caused by the volume variations that occur during breathing [83]. Jayarathna et al. developed a wearable continuous monitoring device, named VitalCore. It is composed of stretchable electroresistive bands (ERBs) based on carbon-black-impregnated polymer in a U-shaped configuration to capture breathing pattern from torso expansion and contraction, ECG electrodes to monitor cardiac activity and accelerometers to detect the body position, all attached to a T-shirt that can be worn during sleep without loss of signal quality [84]. Furthermore, Gargiulo et al. proposed a single electroresistive band, made of conductive silicone tubes, as a wearable contactless sensor to measure cardiac stroke volume, tidal volume and respiratory effort simultaneously during sleep, thus supporting the long-term monitoring of pneumocardiogram (PNCG) [77,78].

A particular class of wearable respiration sensors, which is based on smart textiles, has been the focus of the most recent research in this field. Massaroni et al. developed a smart textile equipped with six piezoresistive sensing elements and showed that, when placed on the pulmonary rib cage, the abdominal rib cage and the abdomen, the measurement system allows recording the respiration-induced movements of the chest wall in a wide frequency range [85]. Issatayeva et al., instead, proposed a fiber-optic-based smart textile for real-time monitoring of breathing rate. The proposed system is composed of two arrays of five fiber Bragg grating sensors embedded into elastic belts, located on 10 different recording sites on the subject’s chest and abdomen, and aims to reconstruct the breathing pattern by converting respiratory movements to strain values [86]. Moreover, Choudhry et al. presented textile-based piezoresistive sensors, which were developed by stitching multifilament conductive threads on the fabric. These sensors were embedded into garments, measuring variations in their resistance due to pressure changes caused by movements of respiratory muscles [87]. Furthermore, Guay et al. made a respiration sensor using multimaterial fibers arranged in a spiral antenna and integrated into textile. The lung volume fluctuations and the textile stretching under the chest movements result in the shift of the operational frequency of the antenna, thus allowing the monitoring of respiration activity. They also showed that the antenna can be used for respiration data transmission to mobile devices via Bluetooth [88].

Finally, in a technical systematic review, Vanegas et al. categorized respiration monitoring systems according to different criteria, including sensor type, respiration parameter and sensor location. They were substantially divided into two categories: wearable and environmental sensors. For both, chest wall movement detection is the most widespread sensing technique, adopted in 60% of the considered studies, while the respiration rate is the most acquired breathing parameter. Furthermore, in the wearable category, fiber-optic sensors are the most used, followed by resistive sensors, accelerometers and capacitive sensors. In the environmental category, instead, the predominant technique is represented by radar sensors. In addition, wearable sensors are usually placed on the chest or the abdomen of the subject, unlike environmental sensors which are placed at a fixed distance from the subject [89].

Very recently, some of the authors presented the novel forcecardiography (FCG) technique for the measurement of the chest wall vibrations induced by the mechanical activity of the beating heart [90]. This technique proved capable of acquiring, in addition to a seismocardiogram-like signal, a novel low-frequency component that seems to carry information on ventricular filling and emptying dynamics. FCG is performed by placing custom-designed force sensors on a subject’s chest wall. FCG sensors are based on force-sensitive resistors (FSRs) that have already proved suitable for muscle contraction monitoring [91] and have enabled the recognition of different hand gestures [92]. Thanks to the wide bandwidth of the FSRs [91], the FCG sensors reasonably appear to be suitable for the measurement of tissue motion originating from many kinds of physiological mechanical events; for this reason, their potential application in respiration monitoring is worth investigating. Indeed, if properly coupled with a subject’s chest, an FCG sensor can measure the force exerted by the ribcage expansions and consequent releases that occur during the breathing acts, thus offering the possibility to simultaneously record a respiration-related signal in addition to the forcecardiogram.

This study aims to demonstrate the suitability of the FCG sensor [90] for accurate, continuous and unobtrusive monitoring of respiration. Experimental tests were performed on seven healthy volunteers, who were asked to perform several respiration cycles while resting on a chair. Simultaneous recordings were acquired from an FCG sensor, an ERB and an ECG monitor. The respiration signal provided by the FCG sensor and the EDR signal extracted from ECG were compared to the ERB signal, which was considered as the benchmark. To this end, statistical analyses were performed on the interbreath intervals estimated from the three acquired signals. The results of this proof of concept show that the FCG respiration signal provides very accurate estimates of the interbreath intervals and clearly outperforms the EDR signal. Due to its ability to acquire information on both respiratory and cardiac activity, the FCG sensor could be effectively used for cardiorespiratory monitoring, but further tests on larger cohorts of subjects are needed to confirm such performances.

## 2. Materials and Methods

### 2.1. Forcecardiography Sensor

**Figure 1:**
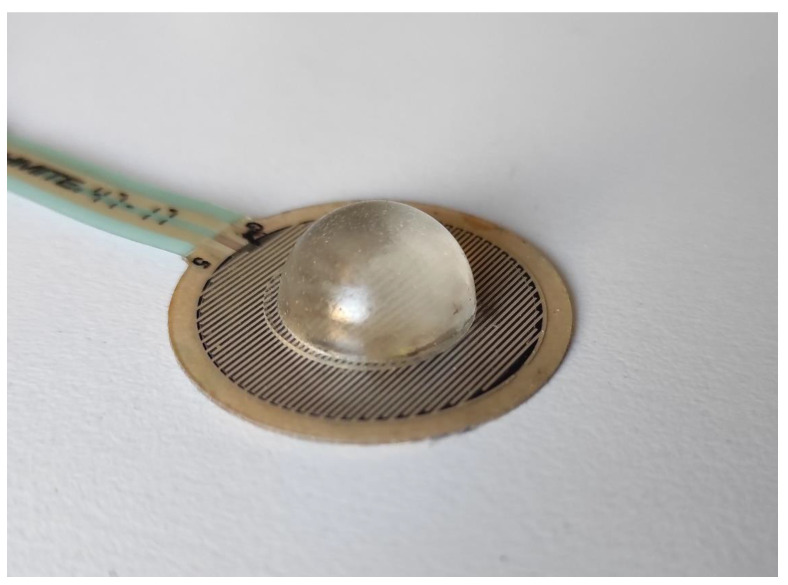
The FCG sensor used in the experimental tests.

The FCG sensor used in this study is depicted in Figure 1. It consists of a force-sensing resistor (FSR03CE, Ohmite Mfg Co, Warrenville, IL, USA) equipped with a dome-shaped mechanical coupler, which ensures a good transduction of the force to the active area of the sensor. The FSR responds to a force applied on its active area by changing its electrical resistance, which must be conveniently transduced into a voltage signal by means of a conditioning circuit [93,94].

In particular, considering that the FSR shows a linear response to the applied force in terms of electrical conductance, a conditioning circuit based on a transimpedance amplifier was used [90,91]. This circuit ensures linearity and minimizes sensor drift by keeping the voltage across the FSR at a constant value [91,93,94]. The FCG sensor was calibrated before measurements in order to obtain the transduction coefficient from voltage to force [90,91].

### 2.2. Electroresistive Band for Respiration Monitoring

**Figure 2:**
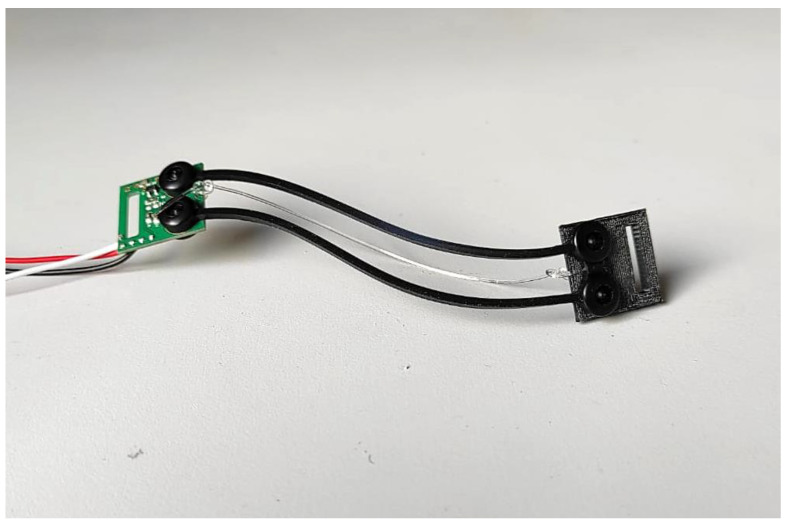
The electroresistive band used in the experimental test.

The assessment of FCG sensor performances in respiration monitoring required the comparison with a reference method. The respiration monitoring method presented in [84], which is based on the use of an ERB applied on the chest of the subject, was adopted as a benchmark. An ERB consists of a stretchable stripe or cord, made of conductive rubber, that increases its electrical resistance when stretched. Hence, it can be used to monitor the increases and decreases in chest circumference that occur during the inhalation and exhalation phases of the respiratory acts. The ERB used in the experimental tests is based on carbon-black-impregnated polymer in a U-shaped configuration and is shown in Figure 2.

### 2.3. Sensor Placement and Measurement Setup

**Figure 3:**
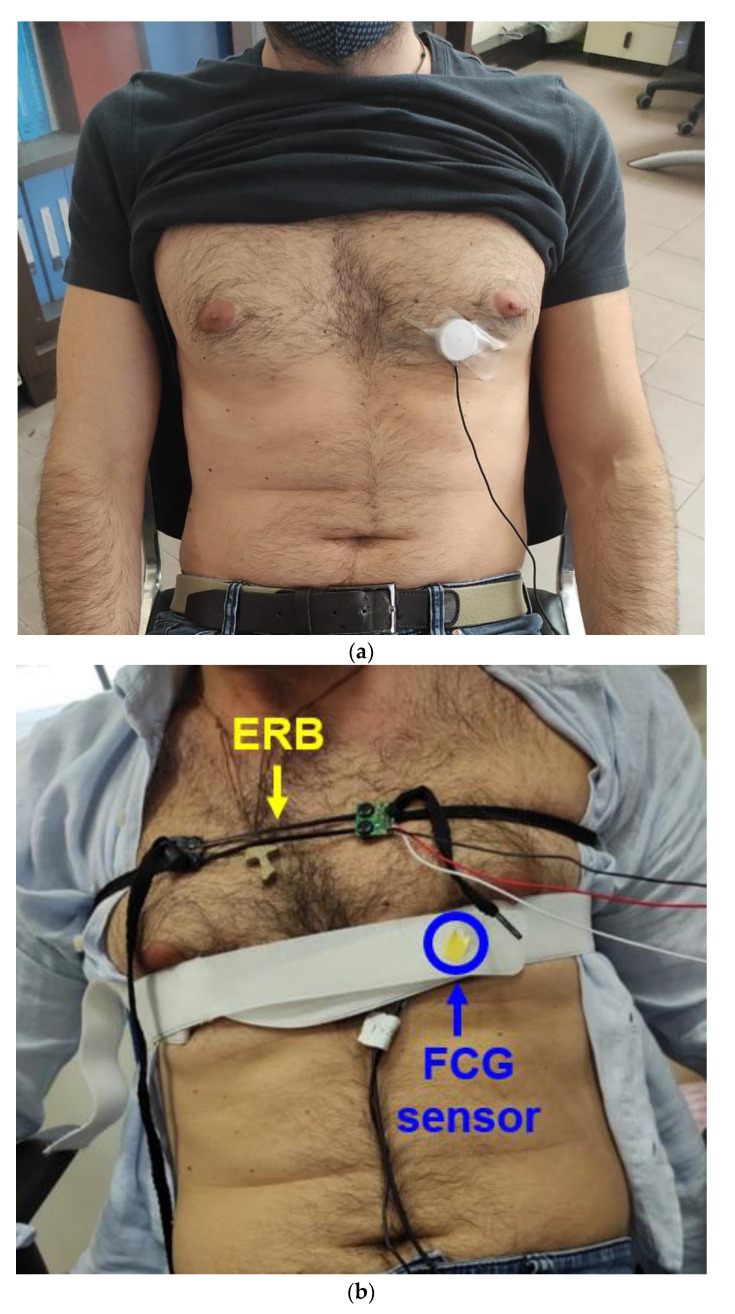
Example of ERB and FCG sensor placement on the chest of subject #7: (**a**) FCG sensor mounted on patient’s chest by means of medical adhesive tape; (**b**) FCG sensor secured on the chest via a belt fastened around the thorax, with the ERB mounted on the upper chest, so as not to interfere with the FCG sensor.

The FCG sensor was placed on the chest of the subject via medical adhesive tape, by first locating the point of maximal impulse (PMI), and then fastened with a belt around the thorax. The ERB for respiration monitoring was mounted on the upper chest of the subject so as not to interfere with the FCG sensor. An ECG lead II was also acquired by means of a WelchAllyn Propaq Encore monitor (Welch Allyn Inc., New York, NY, USA). Figure 3 shows the placement of the FCG sensor and the ERB on subject #7. The signals provided by the ERB, the FCG sensor and the ECG monitor were simultaneously acquired via a National Instrument NI-USB6009 DAQ board, with 13-bit precision and 5 kHz sampling frequency.

The experimental tests were carried out on 7 healthy volunteers (5 males and 2 females, age 33.7 ± 11.5), who signed the informed consent. The subjects comfortably sat on a chair, leaning against the seatback while keeping their back straight. Different acquisitions were performed, where each subject was first asked to breathe in a natural way, then to slightly increase the breathing rate and finally to slow down the respiratory rhythm, so as to obtain measurements of a wide range of breathing rates.

### 2.4. Data Processing and Analysis

The raw signals acquired from the FCG sensor, the ERB and the ECG were first pre-processed to extract the respiration signals, and the peaks related to the inspiratory acts were then located, in order to estimate the interbreath intervals. All processing operations were performed in MATLAB R2017b (The MathWorks, Inc., 1 Apple Hill Drive, Natick, MA, USA).

In particular, both the FCG and ERB signals were low-pass filtered at 0.6 Hz, to maintain the low-frequency components that provide information on the respiratory activity and filter out the spectral components at higher frequencies, which are mainly related to the cardiac activity and to the electronic noise. The rationale for the choice of the cut-off frequency is that the average number of breaths per minute in adult healthy subjects ranges between 12 and 18 [95], corresponding to the 0.2–0.3 Hz frequency range, and thus the cut-off frequency was set by considering a doubled rate (i.e., 0.6 Hz) as a reasonable upper limit, able to filter out the cardiac components. The respiration signal extracted from the raw FCG signal was referred to as R-FCG to avoid confusion with the whole FCG, which also contains information on the cardiac activity.

The EDR signal was extracted from the raw ECG signal by means of the “*BioSigKit*” MATLAB toolbox [96] and was reversed in amplitude to obtain positive peaks corresponding to inspiratory acts, as for the R-FCG and ERB respiration signals. Then, in each of the three respiration signals thus obtained, the positive peaks were located via the MATLAB function “*findpeaks*”, and the interbreath intervals were computed. The inspiratory peaks detected in the R-FCG and EDR signals were compared with those detected in the ERB signals to annotate the number of missed and spurious peaks. The interbreath intervals related to the missed and spurious peaks were discarded for the subsequent statistical analyses. The accuracy and reliability of the FCG sensor in respiration monitoring were assessed by comparing the interbreath interval measures obtained from the R-FCG signals with those obtained via the ERB. To this end, correlation and Bland–Altman analyses were carried out via the MATLAB function “bland-altman-and-correlation-plot” [97]. The same analyses were repeated for the interbreath intervals obtained from the EDR. Finally, the FCG sensor and EDR performances obtained from these analyses were compared.

## 3. Results

**Figure 4:**
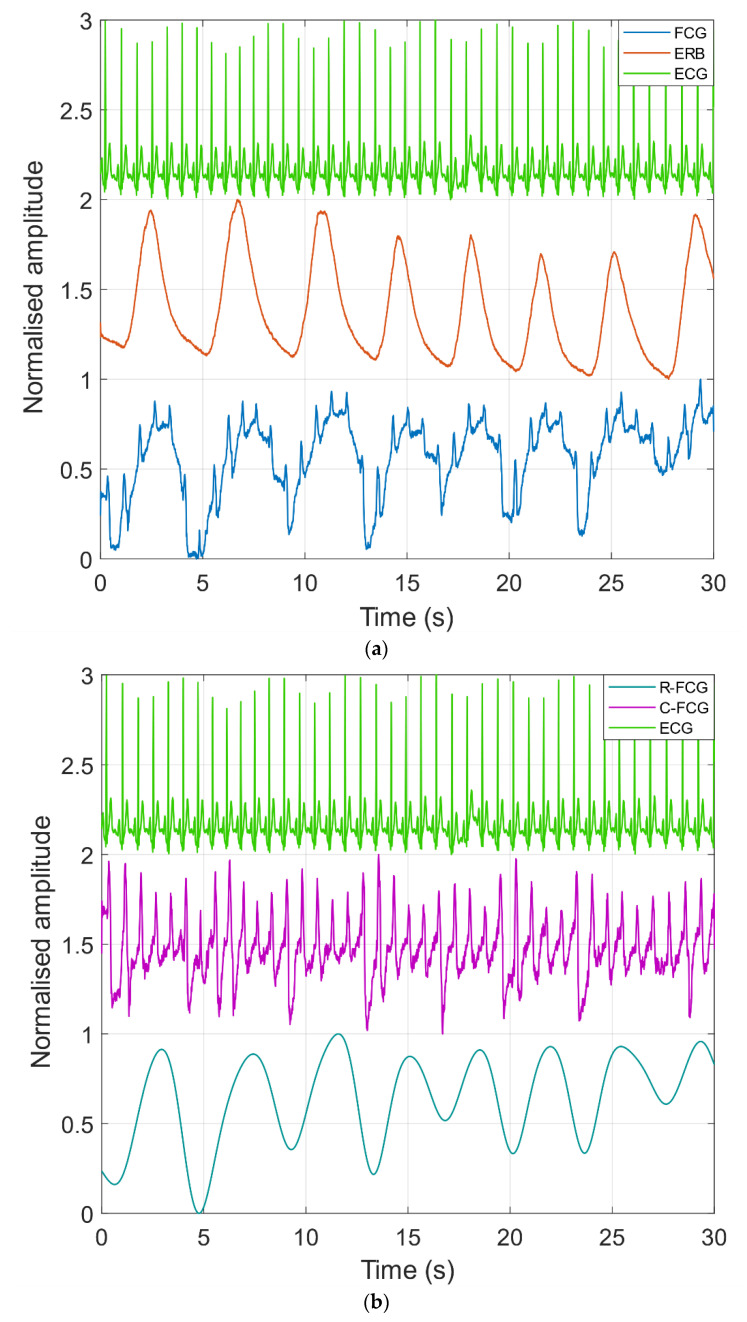
(**a**) An excerpt of raw FCG, ERB and ECG signals acquired from subject #7; (**b**) respiratory (R-FCG) and cardiac (C-FCG) components extracted from FCG signal depicted in panel (**a**).

Figure 4a shows an excerpt of the raw FCG, ERB and ECG signals acquired. In the raw FCG signal, the typical FCG components related to the cardiac activity [90] appear as superimposed to a much larger and slower component, which is related to the respiration. Indeed, Figure 4b depicts the respiratory and the cardiac components extracted from the same FCG signal depicted in Figure 4a.

**Figure 5:**
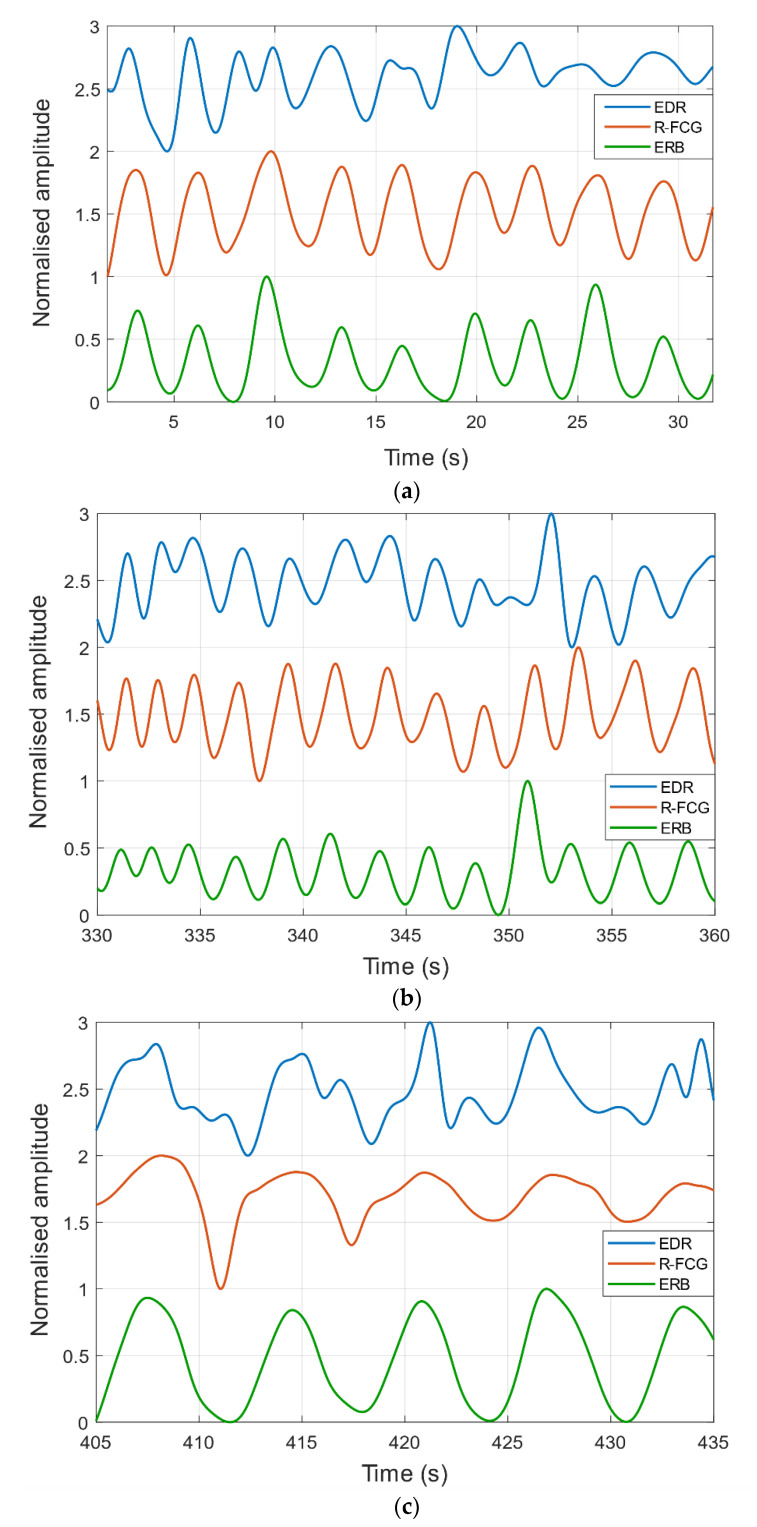
Examples of ERB, R-FCG and EDR signals extracted from data acquired on subject #3: (**a**) quiet breathing; (**b**) forced breathing at higher rate; (**c**) forced breathing at lower rate.

Examples of ERB, R-FCG and EDR signals are shown in Figure 5. In particular, an excerpt from signals acquired on subject #3 during quiet breathing is depicted in panel (a), while excerpts from signals corresponding to forced breathing at higher and lower respiratory rates are shown in panels (b) and (c), respectively. In all three panels, it can be observed that the R-FCG signals featured very similar peaks as compared to the reference ERB signals. The EDR signals, instead, exhibited a much higher variability, which is particularly noticeable in the signals related to the forced slower breathing. Indeed, the EDR clearly presented peaks that turned out to be inconsistently lagged with respect to ERB ones, as well as a number of double peaks corresponding to single ERB peaks, which resulted in a conspicuous number of spurious peak detections.

Table 1 outlines the number of respiratory acts detected per subject in the ERB, R-FCG and EDR signals, along with the number of missed and spurious acts in the R-FCG and EDR signals. A total of 743 respiratory acts were detected in the ERB signal and provided the ground truth to recognize the potential missed or spurious respiratory acts in the R-FCG and EDR signals. No missed respiratory acts were found in the R-FCG signal; i.e., all actual inspiratory peaks were correctly detected, and only eight spurious peaks were misclassified as actual respiratory acts. Hence, the R-FCG scored a sensitivity of 100% and a positive predictive value (PPV) of 98.9%. As opposed to the R-FCG signal, a total of 31 missed respiratory acts were found in the EDR signal, along with 58 spurious respiratory acts, resulting in an overall sensitivity and PPV of 95.8% and 92.5%, respectively.

**Figure 6:**
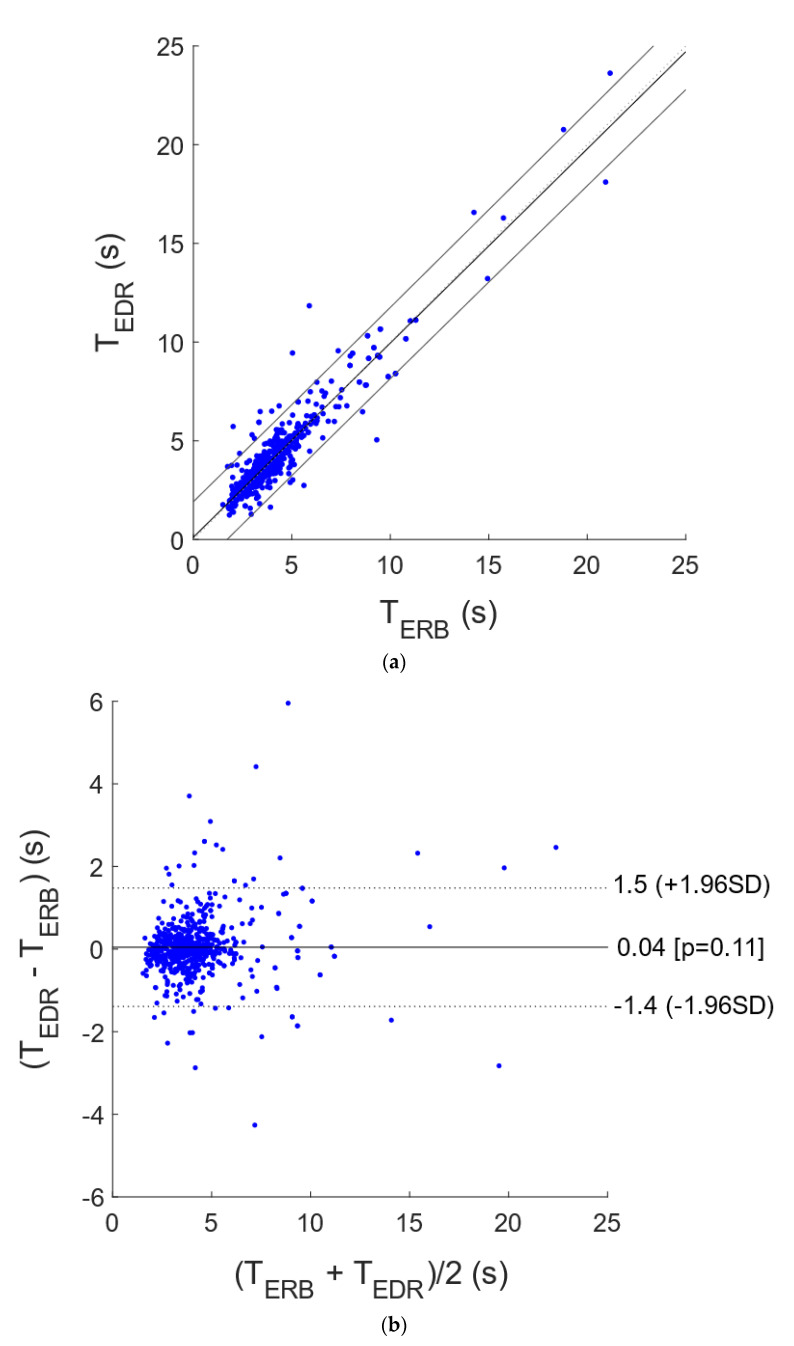
Statistical analyses on inter-respiration intervals estimated from EDR and ERB: (**a**) results of correlation analysis; (**b**) results of Bland–Altman analysis.

**Figure 7:**
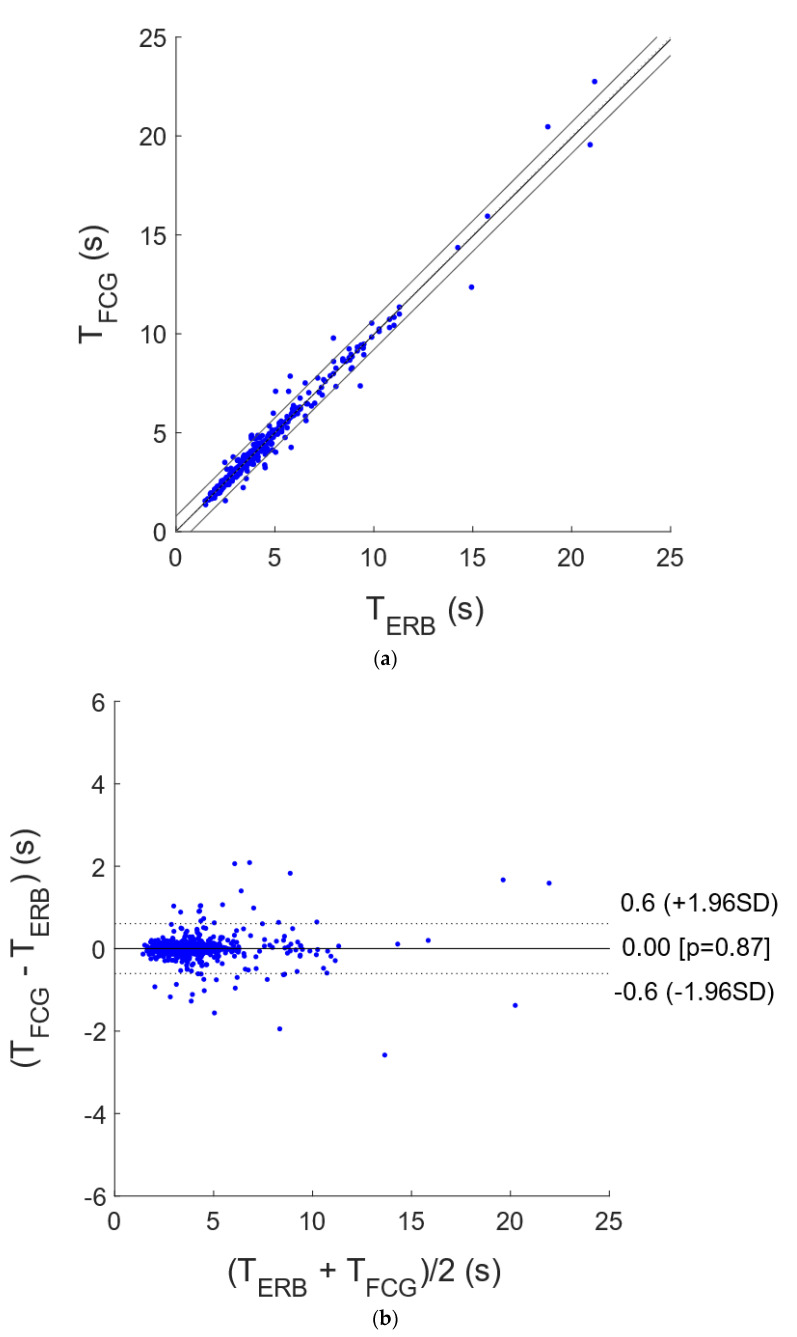
Statistical analyses on inter-respiration intervals estimated from FCG and ERB: (**a**) results of correlation analysis; (**b**) results of Bland–Altman analysis.

The interbreath intervals related to the respiratory acts detected in the ERB, R-FCG and EDR signals were further compared by means of statistical analyses. In particular, the interbreath intervals from R-FCG and EDR were compared with those provided by the ERB via correlation and Bland–Altman analyses. To this end, the intervals related to the missed and spurious respiratory acts were discarded from both the particular signal under test and the reference ERB signal, so as to carry out the analyses only on reliable measurements. The statistical analyses were performed on 743 and 689 interbreath intervals for the R-FCG and EDR signals, respectively, and the results are depicted in Figure 6 and Figure 7. The correlation analysis reported for the R-FCG a slope and intercept of 0.99 and 0.026 s, with an R^2^ value of 0.98, and for the EDR a slope and intercept of 0.98 and 0.11 s, with an R^2^ value of 0.88. The Bland–Altman analysis reported a null bias (*p* = 0.87) with limits of agreement of ±0.61 s for the R-FCG and a bias of 0.040 s (not statistically significant as *p* = 0.11) with limits of agreement of +1.5 and −1.4 s for the EDR.

## 4. Discussion

This study focused on the assessment of the suitability of FCG sensors for accurate, continuous and unobtrusive monitoring of respiration. To this end, simultaneous measurements from an FCG sensor, an ERB and an ECG monitor were acquired on seven healthy subjects at rest. The raw FCG signals showed up as the superimposition of a large, very low frequency component related to the respiratory activity and a smaller component corresponding to the FCG signal [90], which carries information about the cardiac activity. The FCG signal component related to the respiratory activity, referred to as R-FCG, was extracted from the raw FCG signal by means of simple low-pass filtering, which was also applied to the other respiration signals, i.e., EDR and ERB.

The FCG sensor performances for respiration monitoring were assessed by comparing the R-FCG signal with the EDR signal and the ERB signal, which was assumed as the reference. First, the inspiratory peaks were extracted from the three respiration signals, and then both missed and spurious peaks were identified in the R-FCG and EDR signals with respect to the ground truth provided by the ERB signal; finally, the sensitivity and the PPV were computed. In particular, R-FCG exhibited no missed peaks out of 743 actual inspiratory peaks (all subjects) and only 8 spurious peaks, with sensitivity of 100% and PPV of 98.9%, while the EDR exhibited 31 missed peaks and 58 spurious ones, with sensitivity and PPV of 95.8% and 92.5%, respectively. Hence, FCG turned out to be substantially more sensitive and precise than EDR in the detection of inspiratory peaks.

Furthermore, correlation and Bland–Altman analyses were carried out to compare the performances of FCG and EDR for the measurement of interbreath intervals, with respect to the reference measures provided by the ERB. For each signal, only the correctly detected peaks were used for the computation of interbreath intervals and the subsequent statistical analyses. R-FCG and ERB measures exhibited slope and intercept of 0.99 and 0.026, respectively, with an R^2^ value of 0.98, while slope and intercept for EDR and ERB measures were 0.98 and 0.11, respectively, with a substantially lower R^2^ value of 0.88. In addition, both methods exhibited a null bias with respect to the ERB; however, the limits of agreement were more than doubled in the EDR with respect to the FCG (about ±1.5 s compared to ±0.61 s), which demonstrated superior accuracy.

These results of this proof of concept show that monitoring respiration via FCG sensors is feasible and provides accurate detection and measurements of respiratory cycles in subjects at rest. Although no particular problems arose with the two female subjects involved in this study, some issues may possibly arise due to the particular morphology of the breasts. In principle, large breast tissue could cause attenuation of the precordial vibrations, as well as motion artifacts. This hypothetical drawback deserves deeper investigation in larger female cohorts. The possibility of monitoring the respiration and FCG signals simultaneously by means of a single, local, small, unobtrusive, cheap sensor extends the scope of FCG to monitoring multiple vital signs, as well as to the analysis of cardiorespiratory interactions, also paving the way for applications that support the pervasive, continuous, long-term monitoring of cardiorespiratory functions in patients with heart and pulmonary diseases. Moreover, future studies should focus on assessing the viability of FCG-based monitoring in physical activities, such as walking or running, performing heavy work or engaging in sport activities. This would require extensive testing to verify the robustness of FCG regarding motion artifacts, as already observed in accelerometer-based SCG studies [98,99,100,101].

## Figures and Tables

**Table 1:** Respiration acts detected in the ERB, R-FCG and EDR signals. The missed and spurious acts are reported for the R-FCG and EDR signals with reference to the acts detected in the ERB signal.

Subject	Respiration ACTS			Missed ACTS		Spurious ACTS	
	ERB	R-FCG	EDR	R-FCG	EDR	R-FCG	EDR
#1	90	93	95	0	0	3	5
#2	110	111	107	0	7	1	4
#3	177	180	178	0	6	3	7
#4	74	74	75	0	3	0	4
#5	86	87	95	0	3	1	12
#6	76	76	88	0	12	0	24
#7	130	130	132	0	0	0	2
**Total**	743	751	770	0	31	8	58
